# Switching from eltrombopag to hetrombopag in patients with primary immune thrombocytopenia: a post-hoc analysis of a multicenter, randomized phase III trial

**DOI:** 10.1007/s00277-024-05826-5

**Published:** 2024-06-06

**Authors:** Heng Mei, Xiaofan Liu, Yan Li, Hu Zhou, Ying Feng, Guangxun Gao, Peng Cheng, Ruibin Huang, Linhua Yang, Jianda Hu, Ming Hou, Yazhou Yao, Li Liu, Yi Wang, Depei Wu, Xuliang Shen, Jie Jin, Jianmin Luo, Yun Zeng, Xin Zhou, Ruixiang Xia, Zhongxing Jiang, Yuansong Bai, Ting Niu, Renchi Yang, Yu Hu

**Affiliations:** 1 Union Hospital, Tongji Medical College, Huazhong University of Science and Technology, No.1277 Jiefang Avenue, Wuhan, Hubei China; 2 Institute of Hematology and Blood Diseases Hospital, Chinese Academy of Medical Sciences and Peking Union Medical College, No.288 Nanjing Road Heping District, Tianjin, China; 3 West China Hospital, Sichuan University, No.37 Guoxue Alley, Wuhou District, Chengdu, Sichuan China; 4 Affiliated Cancer Hospital of Zhengzhou University, Zhengzhou, China; 5 The Second Affiliated Hospital of Guangzhou Medical University, Guangzhou, China; 6 The First Affiliated Hospital of Air Force Medical University, Xi’an, China; 7 The First Affiliated Hospital of Guangxi Medical University, Nanning, China; 8 The First Affiliated Hospital of Nanchang University, Nanchang, China; 9 The Second Hospital of Shanxi Medical University, Taiyuan, China; 10 Fujian Medical University Union Hospital, Fuzhou, China; 11 Qilu Hospital, Shandong University, Jinan, China; 12 Baoji Central Hospital, Baoji, China; 13 The Second Affiliated Hospital of Air Force Medical University, Xi’an, China; 14 Shaanxi Provincial People’s Hospital, Xi’an, China; 15 The First Affiliated Hospital of Soochow University, Suzhou, China; 16 Heping Hospital Affiliated to Changzhi Medical College, Changzhi, China; 17 The First Affiliated Hospital, Zhejiang University College of Medicine, Hangzhou, China; 18 The Second Hospital of Hebei Medical University, Shijiazhuang, China; 19 First Affiliated Hospital of Kunming Medical University, Kunming, China; 20 Wuxi People’s Hospital, Wuxi, China; 21 The First Affiliated Hospital of Anhui Medical University, Hefei, China; 22 The First Affiliated Hospital of Zhengzhou University, Zhengzhou, China; 23 China-Japan Union Hospital of Jilin University, Changchun, China

**Keywords:** Immune thrombocytopenia, Switching, Thrombopoietin receptor agonist, Eltrombopag, Hetrombopag

## Abstract

While studies have explored the feasibility of switching between various thrombopoietin receptor agonists in treating immune thrombocytopenia (ITP), data on the switching from eltrombopag to hetrombopag remains scarce. This post-hoc analysis of a phase III hetrombopag trial aimed to assess the outcomes of ITP patients who switched from eltrombopag to hetrombopag. In the original phase III trial, patients initially randomized to the placebo group were switched to eltrombopag. Those who completed this 14-week eltrombopag were eligible to switch to a 24-week hetrombopag. Treatment response, defined as a platelet count of ≥ 50 × 10^9^/L, and safety were evaluated before and after the switch. Sixty-three patients who completed the 14-week eltrombopag and switched to hetrombopag were included in this post-hoc analysis. Response rates before and after the switch were 66.7% and 88.9%, respectively. Among those with pre-switching platelet counts below 30 × 10^9^/L, eight out of 12 patients (66.7%) responded, while eight out of nine patients (88.9%) with pre-switching platelet counts between 30 × 10^9^/L and 50 × 10^9^/L responded post-switching. Treatment-related adverse events were observed in 50.8% of patients during eltrombopag treatment and 38.1% during hetrombopag treatment. No severe adverse events were noted during hetrombopag treatment. Switching from eltrombopag to hetrombopag in ITP management appears to be effective and well-tolerated. Notably, hetrombopag yielded high response rates, even among patients who had previously shown limited response to eltrombopag. However, these observations need to be confirmed in future trials.

## Introduction

Primary immune thrombocytopenia (ITP) stands as a noteworthy autoimmune disorder, principally characterized by a transient or enduring decline in platelet count, thereby predisposing patients to an elevated risk of hemorrhagic events [1]. Epidemiologically, the disorder manifests at an annual incidence rate ranging from 2 to 10 cases per 100,000 individuals within the general population [2]. Clinically, ITP often presents with cutaneous and mucosal hemorrhage. However, in severe instances, patients may experience more ominous manifestations, including visceral hemorrhage or even life-threatening intracranial bleeds [1].

The cornerstone of managing ITP is the augmentation of platelet counts to clinically safe levels, thereby mitigating hemorrhagic risks, all while minimizing treatment-related adverse events (TRAEs) and emphasizing health-related quality of life for patients [3, 4]. Traditional first-line treatments typically encompass observation, corticosteroid administration, and intravenous immunoglobulins. However, when first-line treatments either fail to yield desirable outcomes or engender intolerable side-effects, second-line therapeutic modalities such as rituximab, splenectomy, and thrombopoietin receptor agonists (TPO-RAs) are warranted [3, 4]. TPO-RAs have notably revolutionized the treatment paradigm of ITP. By activating thrombopoietin receptors, TPO-RAs trigger the JAK2/STAT5 signaling pathway, thereby promoting the proliferation of megakaryocyte progenitors and enhancing platelet production [5]. Clinical trials indicate that TPO-RAs boast high response rates exceeding 60% [6–9]. As it stands, a range of TPO-RAs, including romiplostim, eltrombopag, avatrombopag, and hetrombopag, have been introduced into clinical practice, thereby broadening the therapeutic arsenal and offering more well-tolerated and effective treatment options for ITP patients [10].

Hetrombopag represents a novel oral TPO-RA, receiving approval from the China National Medical Products Administration for both ITP and aplastic anemia in 2021 [11]. Preclinical investigations have elucidated that hetrombopag shares a similar mechanism of action with eltrombopag. Intriguingly, hetrombopag demonstrates a pharmacological potency approximately 30 times greater than that of eltrombopag [11, 12]. Phase I studies further substantiate its heightened efficacy, revealing that hetrombopag is at least five times more potent in augmenting platelet counts among healthy individuals [13]. Its efficacy and safety profiles for ITP patients have been rigorously evaluated in a randomized, double-blind, placebo-controlled, multicenter phase III trial [9]. Notably, hetrombopag has a significantly lower incidence of hepatotoxicity compared to eltrombopag, suggesting that its lower effective dose may mitigate off-target toxic effects.

Switching between TPO-RAs is becoming an increasingly common treatment strategy in the real-world management of ITP. Existing retrospective studies suggest that patients may maintain or achieve a treatment response when switching to an alternative TPO-RA due to a variety of reasons, including lack of efficacy, patient preference, variable platelet counts, or adverse events (AEs) [14–17]. While these studies documented the successful outcomes of ITP patients switching between different TPO-RAs like eltrombopag, romiplostim, and avatrombopag, no data are currently available on switching from eltrombopag to hetrombopag specifically, warranting further investigation. Given these considerations, the aim of this post-hoc analysis of the hetrombopag phase III trial is to evaluate the outcomes in patients with ITP who switched from eltrombopag to hetrombopag.

## Methods

### Study design and patients

This study utilized post-hoc data derived from a multicenter, phase III, randomized, double-blind, placebo-controlled trial aimed at assessing the efficacy and safety of hetrombopag in adult patients with ITP. The trial was conducted across 33 sites in China and organized into four sequential stages. Stage 1 involved a 10-week, double-blind, placebo-controlled treatment period, wherein participants were randomized to receive either hetrombopag or a placebo. This was followed by stage 2, a 14-week open-label treatment period, during which patients initially assigned to the placebo group were switched to eltrombopag, commencing with a daily dose of 25 mg that could be adjusted up to a maximum of 75 mg. Patients who completed this 14-week eltrombopag treatment were eligible to directly switch to an additional 24-week hetrombopag treatment in Stage 4. The recommended starting dose for hetrombopag was 5 mg per day, based on prior data indicating minimal platelet count fluctuations at this dose, though investigators had the discretion to adjust the starting dosage as needed (range: 2.5 to 7.5 mg per day). Stage 3 comprised a dose tapering period lasting up to six weeks, culminating in medication withdrawal. Detailed methodologies and findings from stages 1 through 4 for the hetrombopag group have been previously reported [9, 18]. The primary focus of this post-hoc analysis is to investigate the patient response when switching from eltrombopag to hetrombopag during the ITP treatment.

This post-hoc analysis is grounded in data acquired from the original phase III clinical trial, which adhered to the ethical principles delineated in the Declaration of Helsinki as well as Good Clinical Practice guidelines. Ethical approval for the initial trial was granted by the Institutional Review Boards at each participating site. All patients involved provided written informed consent prior to their enrollment. The trial was registered on ClinicalTrials.gov under the identifier NCT03222843.

### Assessments and outcomes

Platelet counts were measured bi-weekly during the 14-week treatment phase with eltrombopag and at four-week intervals during the 24-week treatment phase with hetrombopag. Treatment response was defined as a platelet count of ≥ 50 × 10^9^/L. Various efficacy outcomes were evaluated, including the proportion of patients exhibiting at least one treatment response; those attaining a platelet count of ≥ 30 × 10^9^/L on at least once; platelet counts at each scheduled visit; and the patient requiring protocol-defined rescue therapy, which was determined by the clinical judgment of investigators to be either platelet transfusion or intravenous immunoglobulin. Furthermore, the study evaluated the maximum continuous duration of response, total duration of response, and bleeding instances as per the World Health Organization (WHO) bleeding scale.

AEs were continuously monitored throughout the treatment periods. Comprehensive clinical assessments were carried out at every scheduled study visit, encompassing clinical laboratory evaluations, physical examinations, electrocardiograms, ophthalmological examinations, and bone marrow biopsies. AEs were codified according to the preferred terms of the Medical Dictionary for Regulatory Activities, version 22.0.

### Statistical analyses

All statistical analyses employed in this study were descriptive in nature. Continuous variables were presented as medians and ranges, while categorical variables were denoted as frequencies and percentages. The temporal trends in median platelet counts and platelet response rates were visually represented through line graphs, plotted against the weeks of scheduled visits during both the eltrombopag and hetrombopag treatment phases. All statistical computations were executed using SAS software, version 9.4 (SAS Institute).

## Results

### Patients

**Fig. 1:**
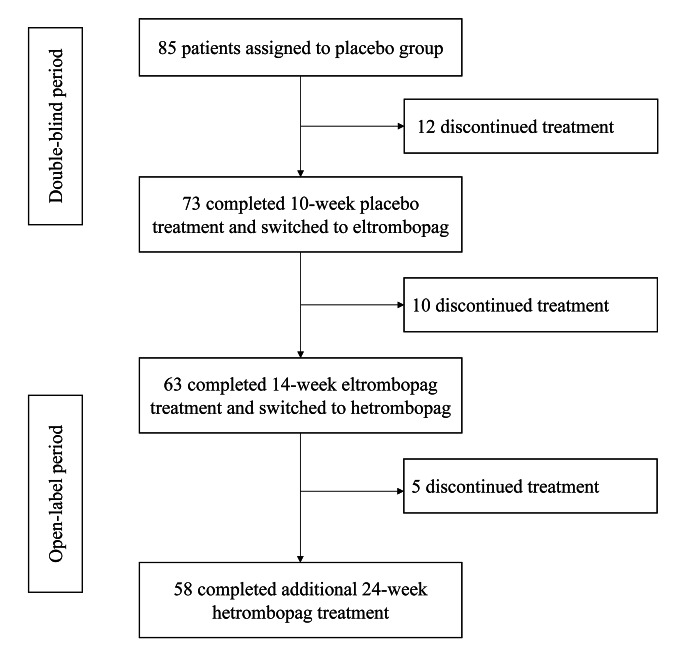
Study design and patient flowchart

In the study cohort, 63 patients (74.1%) successfully completed the initial 24-week treatment protocol (a 10-week placebo phase followed by a 14-week eltrombopag regimen). These patients subsequently switched to a predefined 24-week treatment period with hetrombopag (Fig. 1). At enrollment, the median age of these patients was 43 years (range, 18 to 70), and 47 (74.6%) patients were female. A majority of the patients (63.5%) had been grappling with ITP for three years or more. The median baseline platelet count prior to the switch to hetrombopag was 69.0 × 10^9^/L (range, 6.0 to 278.0 × 10^9^/L). Remarkably, one-third of these patients (33.3%) had failed to attain a sustained platelet response under the eltrombopag treatment. At the time of switching, 4 patients (6.3%) received eltrombopag 25 mg every other day, 14 patients (22.2%) received 25 mg daily, 21 patients (33.3%) received 50 mg daily, and 17 patients (27.0%) received 75 mg daily (Table 1).

**Table 1:** Baseline characteristics of patients and dose levels

Variables	All (*N* = 63)
Age, median (range), years	43 (18, 70)
Female, n (%)	47 (74.6)
Baseline platelet count, median (range), ×10^9^/L	69.0 (6.0, 278.0)
Baseline platelet count (×10^9^/L), n (%)	
< 10	6 (9.5)
≥ 10-<30	6 (9.5)
≥ 30-<50	9 (14.3)
≥ 50-<100	22 (34.9)
≥ 100	20 (31.7)
Time since first ITP diagnosis, years, n (%)	
≥ 0.5-<1	10 (15.9)
≥ 1-<3	13 (20.6)
≥ 3-<5	11 (17.5)
≥ 5	29 (46.0)
Prior splenectomy, n (%)	0
Bleeding (WHO bleeding scale), n (%)	
No bleeding	56 (88.9)
Grade 1	7 (11.1)
Dose of eltrombopag at the time of switching, n (%)	
25 mg, qod	4 (6.3)
25 mg, qd	14 (22.2)
50 mg, qd	21 (33.3)
75 mg, qd	17 (27.0)
Others	7 (11.1)
Final dose of hetrombopag	
2.5 mg, qod	2 (3.2)
2.5 mg, qd	5 (7.9)
3.75 mg, qd	12 (19.0)
5 mg, qd	16 (25.4)
7.5 mg, qd	28 (44.4)

ITP: immune thrombocytopenia; WHO: World Health Organization

### Efficacy outcomes after switching

As of the data cutoff date on November 19, 2020, 58 patients (92%) successfully completed the hetrombopag treatment protocol, with a median exposure duration of 169.0 days (range, 53.0 to 176.0). Among these patients, 28 (44.4%) were administered the final dosage of 7.5 mg daily, while the final dosage was 5 mg daily for 16 patients (25.4%). Notably, a high proportion of patients, 56 out of 63 (88.9%), exhibited a platelet response following the switch from eltrombopag to hetrombopag. Eight out of 12 patients (66.7%) with a pre-switching platelet count below 30 × 10^9^/L, and eight out of nine patients (88.9%) with a pre-switching platelet count between 30 × 10^9^/L and 50 × 10^9^/L achieved a platelet response post-switching.

**Fig. 2:**
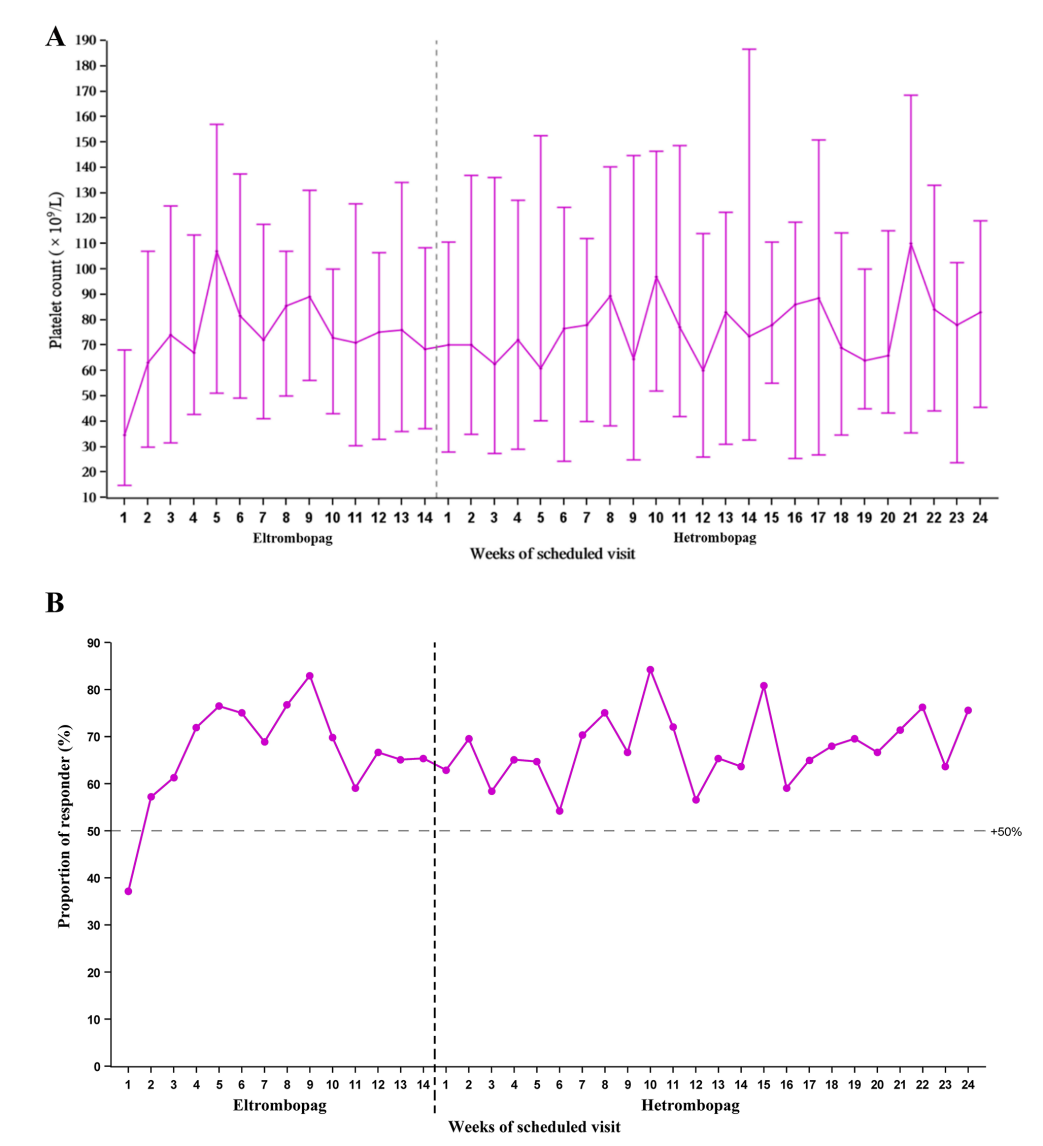
Temporal treatment response: (**A**) Median platelet counts during eltrombopag and hetrombopag treatment; (**B**) Proportion of patients achieving platelet response

The median platelet count ascended to 70 × 10^9^/L within two weeks post-switching and consistently fluctuated between 62.5 × 10^9^/L and 110 × 10^9^/L during the 24-week hetrombopag treatment period, corroborating the maintenance of a stable platelet response (Fig. 2). The median maximum continuous duration and the total duration of the platelet response were 78 days (range, 8 to 165) and 104 days (range, 8 to 165), respectively, after the switch to hetrombopag. Furthermore, five (7.9%) patients required protocol-defined rescue therapy, and three (4.8%) experienced bleeding symptoms, all of which were classified as grade 1 according to the WHO bleeding scale.

### Adverse events

The overall incidence of TRAEs was 50.8% during the eltrombopag treatment, compared to 38.1% during the hetrombopag treatment. The most common TRAEs include platelet counts increased (9.5% during the eltrombopag treatment vs. 7.9% during the hetrombopag treatment), alanine aminotransferase increased (9.5% vs. 1.6%), unconjugated bilirubin increased (7.9% vs. 3.2%), and aspartate aminotransferase increased (6.3% vs. 3.2%). In both treatment stages, seven patients (11.1%) either interrupted the study treatment or required dose adjustments due to the onset of AEs (Table 2).

**Table 2:** Treatment-related adverse events (TRAEs) of any severity occurring at least 5% of patients and moderate-to-severe TRAEs occurring in at least one patient

TRAEs, *n* (%)	Eltrombopag treatment (*N* = 63)							Hetrombopag treatment (*N* = 63)			
	Mild		Moderate	Severe	All			Mild	Moderate	Severe	All
Any TRAE	25 (39.7)		6 (9.5)	1 (1.6)	32 (50.8)			20 (31.7)	4 (6.3)	0	24 (38.1)
Platelet count increased	6 (9.5)		0	0	6 (9.5)			4 (6.3)	1 (1.6)	0	5 (7.9)
ALT increased	4 (6.3)		2 (3.2)	0	6 (9.5)			1 (1.6)	0	0	1 (1.6)
AST increased	2 (3.2)		2 (3.2)	0	4 (6.3)			2 (3.2)	0	0	2 (3.2)
UCB increased	4 (6.3)		1 (1.6)	0	5 (7.9)			2 (3.2)	0	0	2 (3.2)
CPK-MB increased	0		1 (1.6)	0	1 (1.6)			3 (4.8)	0	0	3 (4.8)
Bacterial test positive	1 (1.6)		1 (1.6)	0	2 (3.2)			1 (1.6)	0	0	1 (1.6)
Body weight gain	0		0	0	0			0	1 (1.6)	0	1 (1.6)
Gait disturbances	0		1 (1.6)	0	1 (1.6)			0	0	0	0
Drug-induced liver injury	1 (1.6)		1 (1.6)	0	2 (3.2)			0	0	0	0
Liver disorders	0		1 (1.6)	0	1 (1.6)			0	0	0	0
Urethritis	0		0	1 (1.6)	1 (1.6)			3 (4.8)	0	0	3 (4.8)
Urinary tract infection	1 (1.6)		0	1 (1.6)	2 (3.2)			0	0	0	0
Proteinuria	0		0	0	0			0	1 (1.6)	0	1 (1.6)
Insomnia	0		0	0	0			0	1 (1.6)	0	1 (1.6)

TRAE: treatment-related adverse event; ALT: alanine aminotransferase; AST: aspartate aminotransferase; UCB: unconjugated bilirubin; CPK-MB: creatinine phosphate kinase - muscle and brain

## Discussion

In the evolving landscape of ITP management, switching between TPO-RAs is progressively recognized as a pragmatic treatment strategy [19]. Our post-hoc analysis contributes insights into this practice by providing data on the sequential treatment of ITP, specifically a 14-week course of eltrombopag followed by a 24-week course of hetrombopag. Our findings corroborate the efficacy of switching from eltrombopag to hetrombopag, which also substantiate the absence of cross-resistance between these two TPO-RAs. To the best of our knowledge, this constitutes the first dataset on TPO-RA switching derived from a clinical trial setting, thereby filling a critical gap in the existing literature and offering robust evidence to inform future clinical practice.

TPO-RAs have garnered recognition for their efficacy and tolerability in the management of ITP [5]. However, clinical challenges persist as some patients fail to benefit from a specific TPO-RA due to inefficacy or adverse reactions. Prior case reports by Aoki et al. [20] and D’Arena et al. [17] has highlighted the feasibility and potential benefits of switching between TPO-RAs like eltrombopag and romiplostim, suggesting a lack of cross-resistance and different efficacy profiles between these agents. These observations are underpinned by the distinct molecular and mechanistic characteristics of TPO-RAs. For instance, romiplostim directly competes with endogenous thrombopoietin for receptor binding, while eltrombopag interact with the transmembrane domain of the TPO receptor [5, 21]. This leads to subtly different downstream signaling pathways, such as a greater activation of the AKT pathway by romiplostim and more Janus-kinase signal transducer and activator of transcription (JAK–STAT) activation by eltrombopag [22, 23]. These intricate mechanistic differences are pivotal in understanding the quality of megakaryocyte activation and could explain the observed variability in responses to TPO-RAs. Consistent with this, a pooled review of 18 retrospective data on 401 patients revealed that more than 75% of patients maintained or achieved a platelet response when switched between eltrombopag and romiplostim, irrespective of the switch’s direction [19]. Beyond the well-established agents romiplostim and eltrombopag, a retrospective observational study revealed that avatrombopag, a newer TPO-RA, demonstrated efficacy in patients who had previously been treated with either romiplostim or eltrombopag [15]. Concurrently, a prospective, open-label phase IV study is underway in the United States to further assess the feasibility of avatrombopag following eltrombopag or romiplostim in adults with ITP [24]. Complementing these findings, our own analysis indicated that a substantial majority (88.9%) of patients exhibited a response when switched from eltrombopag to hetrombopag. Collectively, these results advocate for TPO-RA switching as a potentially effective and personalized treatment strategy in the management of ITP.

In this post-hoc analysis, our findings suggest that hetrombopag may present certain advantages over eltrombopag in the management of ITP. Particularly noteworthy is the robust platelet response observed after switching to hetrombopag: 66.7% of patients with pre-switching platelet counts below 30 × 10^9^/L and 88.9% with counts between 30 × 10^9^/L and 50 × 10^9^/L exhibited a platelet response. Although both agents operate via a similar mechanistic framework, binding to the transmembrane domain of the thrombopoietin receptor and triggering specific phosphorylation cascades, the pharmacological potency of hetrombopag appears to be substantially greater. Preclinical studies suggest it is up to 30 times more potent than eltrombopag in promoting cellular proliferation [11, 12]. Furthermore, a phase I study has shown hetrombopag to be at least five times more efficacious than eltrombopag in elevating platelet counts in healthy individuals [13]. The underlying structural modifications in hetrombopag not only enhance its pharmacological activity but also seem to mitigate toxic side effects to a certain extent when compared to eltrombopag [9]. Thus, our study adds compelling evidence to the evolving narrative that hetrombopag could be an optimized therapeutic option in the TPO-RA arsenal for treating ITP. Head-to-head randomized controlled trials are warranted to confirm these findings.

In the realm of safety considerations, our study demonstrated a numerically reduction in the incidence of TRAEs when patients were switched from eltrombopag to hetrombopag (50.8% vs. 38.1%). Importantly, no severe AEs were recorded during hetrombopag administration. This stands in contrast to the documented side effects associated with other approved TPO-RAs, such as romiplostim, eltrombopag, and avatrombopag, where incidences of thromboembolic events, hepatotoxicity, cataracts, prolonged QT intervals, and myelofibrosis have been noted [5]. However, these AEs were rarely observed in our study, which was consistent with our previous findings [9, 18]. Previous research has shown that patients intolerant to one TPO-RA could successfully switch to another, facilitated by the absence of overlapping AEs [19]. Our findings lend support to the idea that hetrombopag could offer a more tolerable alternative for patients who experience AEs with other TPO-RAs.

This study has some limitations. Firstly, previous studies have cited multiple reasons for switching TPO-RAs in ITP patients, including lack of efficacy, patient preference, platelet count fluctuations, and AEs. For those who experienced suboptimal efficacy with their initial TPO-RA, a lower response rate was commonly observed [19]. However, the design of our study mandated that all participants switch from eltrombopag to hetrombopag, leaving the real-world rationale and feasibility for such a switch unexplored. Secondly, the non-randomized nature of our study precludes any conclusions about the natural course of platelet count changes in patients who might have continued on eltrombopag. Consequently, we cannot definitively state whether the observed improvements were exclusively attributable to hetrombopag or might have occurred if eltrombopag therapy had been sustained. Further randomized controlled trials are essential to validate our findings.

## Conclusion

In summary, our post-hoc analysis substantiates that switching from eltrombopag to hetrombopag in the treatment of ITP is both effective and well-tolerated. Specifically, hetrombopag presents a viable therapeutic option in scenarios where eltrombopag yields limited or suboptimal responses, or when AEs necessitate a change in treatment. These findings underscore the potential utility of hetrombopag as a robust alternative in ITP management. Nonetheless, these observations need to be confirmed in future trials.

## Data Availability

The data that support the findings of this study are available from the corresponding author upon reasonable request.

## Declarations

### Clinical trial registration

The study was registered at clinicaltrials.gov with the identifier NCT03222843.
